# Change of the active bacteria mediating HCO_3_^-^-fixation in biological soil crusts using DNA-based stable isotope probing

**DOI:** 10.1016/j.isci.2026.115898

**Published:** 2026-04-28

**Authors:** Yucheng Xie, Huilin Li, Tingting Teng, Dayi Zhang

**Affiliations:** 1 Key Laboratory of Groundwater Resources and Environment Ministry of Education, Jilin University, Changchun 130021, P.R. China; 2 College of New Energy and Environment, Jilin University, Changchun 130021, P.R. China; 3 Key Laboratory of Regional Environment and Eco-restoration, Ministry of Education, Shenyang University, Shenyang 110044, P.R. China

**Keywords:** isotope chemistry, microbiology, soil biology, soil science

## Abstract

Biological soil crusts (BSCs) are important primary producers in drylands. Autotrophs therein are typically dormant in dry seasons and revive in wet seasons. However, active members of dissolved bicarbonate after revival remain underexplored. Here, H^13^CO_3_^−^ was used to determine the active carbon-fixers in BSCs and underlying soils in the resuscitation (RP, 0–7 days) and sustained hydration (SHP, 7–14 days) phases. In the SHP, BSCs had higher nutrient levels and biomass than in the RP. *Blastococcus mobilis*, *Modestobacter altitudinis*, *Scytonema hyalinum*, and some unclassified strains were identified as HCO_3_^−^-fixers based on DNA stable isotope probing (DNA-SIP). Precisely, photoautotrophs and chemoautotrophs were the main HCO_3_^−^-fixers of BSCs in the RP and SHP, respectively. Additionally, ^13^C-transformers were enriched in the underlying soils and related to nutrient cross-feeding. Our findings unveiled bacterial HCO_3_^−^-fixers and their dynamics in BSCs reviving from dormancy, providing clues for the mechanisms of inorganic carbon-fixation in drylands.

## Highlights

• First determination of bicarbonate fixers in biocrusts using DNA-SIP
• *B. mobilis*, *M. altitudinis*, and *S. hyalinum* were newly identified as carbon fixers in BSCs
• Dynamic bicarbonate fixers potentially changed from photoautotrophs to chemoautotrophs

## Graphical abstract

**Figure:**
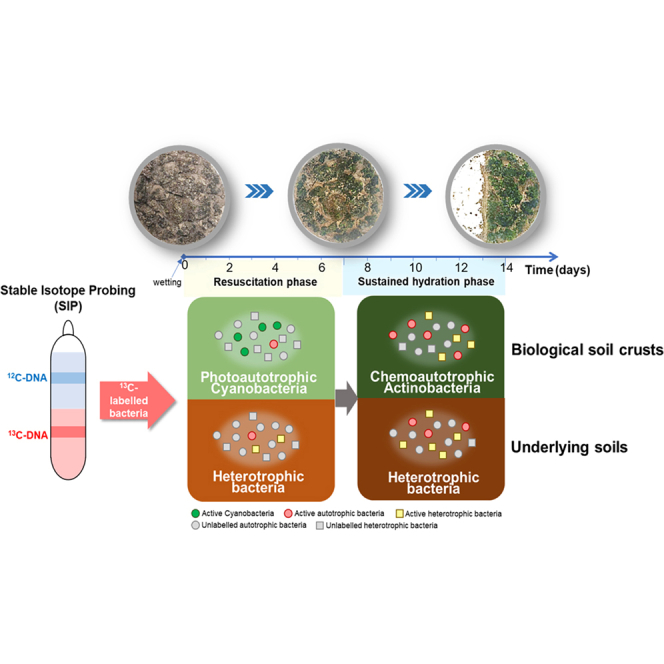

## Introduction

Biological soil crusts (BSCs) are critical landscape units on dryland soil surfaces, forming an ecosystem composed of autotrophic producers (cyanobacteria, microalgae, lichens, mosses, and so forth), consumers (protozoa, microarthropods, and other soil microfauna), and decomposers (bacteria, fungi, archaea, and so forth).^1^ They serve ecological importance for biogeochemical cycling in drylands.^2^^,^^3^ BSCs constitute a substantial portion of primary production in drylands^4^ and play an important role in the worldwide carbon cycle,^5^^,^^6^ owing to the inorganic carbon assimilation processes by autotrophic microorganisms,^7^^,^^8^ Although microorganisms in drylands are found normally dormant due to extreme conditions, such as drought, high radiation, or cold,^9^^,^^10^ they can rapidly switch between dormant and vigorous states which depend on soil hydrothermal conditions.^11^^,^^12^ Precipitation events or transient moisture (such as early morning dewfall) are widely recognized as critical factors for many key biogeochemical processes in drylands, especially inorganic carbon fixation by photosynthetic bacteria or algae.^13^^,^^14^^,^^15^

In many semi-arid ecosystems, dormant microorganisms in BSCs might be resuscitated intensively during the wet season and maximize energy synthesis in the relatively sustained hydrated environments that are likely to last for more than a month.^15^^,^^16^ In addition, soil moisture can directly drive the dissolution of soil inorganic carbon, especially for HCO_3_^−^, which is the most abundant form for microbial inorganic carbon fixation besides CO_2_ under circumneutral pH conditions in drylands.^17^^,^^18^ Among the known inorganic carbon fixation pathways for microbial HCO_3_^−^-fixation, 3-hydroxypropionate (3HP) and 3-hydroxypropionate/4-hydroxybutyrate (3HP4HB) cycles are the major ones.^18^ In addition, although HCO_3_^−^-utilization is not essential in biosynthesis or anaplerotic reactions in other pathways (Calvin-Benson-Bassham (CBB), reductive citric acid (rTCA), and dicarboxylate/4-hydroxybutyrate (DC4HB) cycles), prior studies have reported that HCO_3_^−^ could be a complementary substrate to CO_2_.^18^^,^^19^^,^^20^ Notwithstanding, as most autotrophic species possess a limited carbon fixation pathway,^21^ the carbon fixation mechanisms of BSCs are largely shaped by microbial composition. Previous studies have reported the distinct carbon fixation mechanisms in BSCs under different hydrothermal conditions using metagenomic sequencing^22^^,^^23^; however, there remains a lack of direct experimental evidence on these carbon fixers in BSCs, particularly HCO_3_^−^-fixers that recover from dormancy after hydration and play important roles in the dryland carbon cycle.

Inorganic carbon fixers in BSCs consist mainly of photoautotrophs and chemoautotrophs. Among them, oxygenic photoautotrophic microbes serve as the dominant primary producers in BSCs,^24^^,^^25^ mainly including bacterial Cyanobacteria, Alphaproteobacteria, and Actinobacteria, and many photoautotrophic eukaryotes (such as green algae) based on the diversities of RubisCO and Chlorophyll synthesis genes in BSCs.^9^^,^^26^ Additionally, despite the relatively marginal contribution of chemosynthesis to the increase in total global primary productivity,^27^ many studies have reported the key roles of chemoautotrophs, such as bacterial Actinobacteria, AD3, and WPS-2, in maintaining soil microbial diversity in drylands.^11^^,^^28^^,^^29^ The rapid resuscitation of bacteria in BSCs, particularly Cyanobacteria, from dormancy upon hydration has been widely reported.^30^^,^^31^ However, the knowledge about the changes in inorganic carbon-fixers in BSCs from depleted to sustained hydration conditions remains unknown.

Approaches identifying functional microorganisms have been evolving in recent decades. Traditional cultivation methods can only obtain a limited number of microorganisms, as over 99% of them remain uncultivated; gene sequencing or metagenomes have notably expanded our knowledge of these uncultivated members, facing challenges to confirm their ecological functions.^32^^,^^33^ DNA stable isotope probing (DNA-SIP) is a cultivation-independent approach providing a comprehensive understanding of the active functional members and their ecological functions based on the incorporation of stable-isotope labeled compounds into DNA.^34^^,^^35^^,^^36^ DNA-SIP has successfully explored inorganic carbon fixers and tracked carbon flows in some soil and sediment ecosystems.^37^^,^^38^^,^^39^ In BSCs, DNA-SIP with ^15^N_2_ or H_2_^18^O has identified the active diazotrophs^34^^,^^35^ or bacteria in some specific scenarios.^40^ However, DNA-SIP has not been previously applied to identify the active inorganic carbon fixers in BSCs.

In this study, to determine the active bacteria responsible for HCO_3_^−^-assimilation and their dynamics in BSCs from depleted to sustained hydration, we conducted a DNA-SIP microcosm experiment with ^13^C-HCO_3_^-^ using BSCs collected from a typical semi-arid region on the Qinghai-Tibetan Plateau. We aimed to identify the active bacteria responsible for HCO_3_^−^-fixation, uncover the composition dynamics of the active HCO_3_^−^-fixation bacteria under different hydration conditions, and reveal the HCO_3_^−^-assimilation mechanisms in BSCs. Our findings can provide deeper insights into the microbial utilization of dissolved bicarbonate in BSCs, offering additional information on the transformation and fate of endowed inorganic carbon in drylands.

## Results

### Physicochemical variables of topsoils along incubation

**Figure 1:**
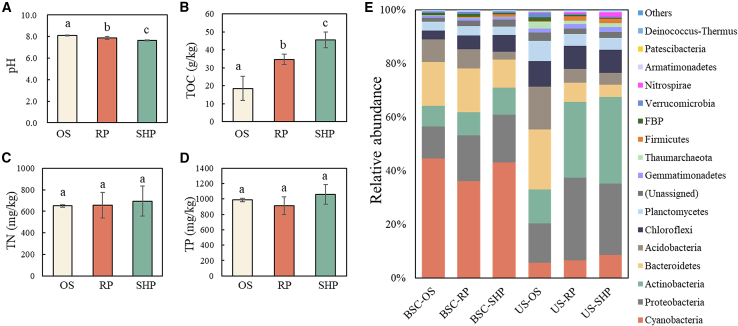
The physicochemical characteristics and microbial information of topsoil over different periods (A) pH, (B) TOC, (C) TN, (D) TP in the OS, RP, and SHP. (E) Bacterial community structures of BSCs and US in OS, RP, and SHP. US refers to the underlying soil. OS represents the original sample. SP represents the resuscitation phase, and SHP represents the sustained hydration phase.

Hydration significantly promoted the growth of BSCs. In particular, mosses started germination at the end of the RP phase and became remarkably flourishing in the SHP phase, showing a delayed response of dormant moss spores to moisture and higher BSC biomass in the SHP than the RP phase (Figure S1). Key physicochemical variables of topsoils in different phases are shown in Figure 1. More precisely, pH values decreased remarkably with incubation time (OS: 8.1, RP: 7.9, SHP: 7.7), while TOC increased (OS: 18.5 g/kg, RP: 34.7 g/kg, SHP: 45.6 g/kg, *p* < 0.05, Figures 1A and 1B). For other nutrients, TN (average 667.2 mg/kg) and TP (average 986.1 mg/kg) did not change significantly (*p* > 0.05), whereas NH_4_^+^-N, NO_3_^−^-N, and AP showed a notable increase along incubation (*p* < 0.05, Figure S2).

### Bacterial communities of BSCs and US

A total of 88,175 clean reads were detected, and 14,032 OTUs were assembled, belonging to 33 bacterial phyla (Figure 1E). Among them, predominate bacterial taxa (>5%) in BSCs included Cyanobacteria (OS: 44.7%, RP: 36.3%, SHP: 43.2%, same as follows), Proteobacteria (11.9%, 17.1%, and 17.7%), Bacteroidetes (16.2%, 16.3%, and 10.4%), Actinobacteria (7.7%, 8.6%, and 10.1%), and Acidobacteria (8.6%, 7.2%, and 3.0%). The compositions of major bacterial taxa were similar between BSCs and US, but their relative abundance was different. Briefly, Actinobacteria was the predominant one in US (12.8%, 28.3%, and 32.3%), followed by Proteobacteria (14.6%, 30.8%, and 26.6%), Bacteroidetes (22.3%, 7.1%, and 4.7%), Acidobacteria (16.0%, 5.2%, and 4.4%), Chloroflexi (9.6%, 8.6%, and 8.6%), and Cyanobacteria (5.8%, 6.6%, and 8.6%). Bacterial community structures in both BSCs and US did not exhibit significant change between the RP and SHP phases (*p* > 0.05, Figure S3).

### Active bacteria responsible for HCO_3_^−^-fixation and carbon cycling in BSCs and US in the resuscitation phase

**Figure 2:**
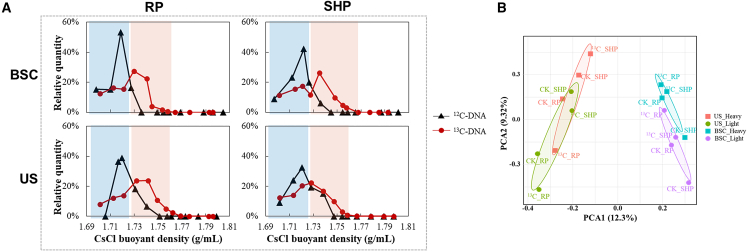
DNA ultracentrifugation information (A) Quantity of DNA in different fractions collected from ^12^C-HCO_3_^-^ and ^13^C-HCO_3_^-^ treatments against buoyant density (BD). (B) Principal component analysis (PCA) score plot of bacterial communities in light and heavy-DNA fractions from BSCs and US (the underlying soils).

^12^C- and ^13^C-labelled DNA were separated into twelve DNA fractions after isopycnic ultracentrifugation. The relative abundance of DNA from ^12^C-HCO_3_^-^-labelled samples peaked in Fraction 10 with the BD values ranging from 1.7186 to 1.7219 g/mL; whereas in ^13^C-HCO_3_^-^-labelled treatments, DNA mostly accumulated in Fraction 9 (BD values: from 1.7274 to 1.7317 g/mL), except for BSC samples (SHP phase) where ^13^C-DNA enriched in Fraction 8 with a BD value of 1.7371 g/mL. Detailed BD values for stratifying each sample are presented in Table S1. Generally, the fractions with BD values >1.7274 g/mL were assigned to heavy DNA, while those with BD values <1.7274 g/mL were assigned to light DNA (Figure 2A). Moreover, the PCA score plot (Figure 2B) exhibited a notable separation between bacterial communities from the light- and heavy-DNA fractions in either BSCs or US, showing the significant difference in bacterial communities.

**Figure 3:**
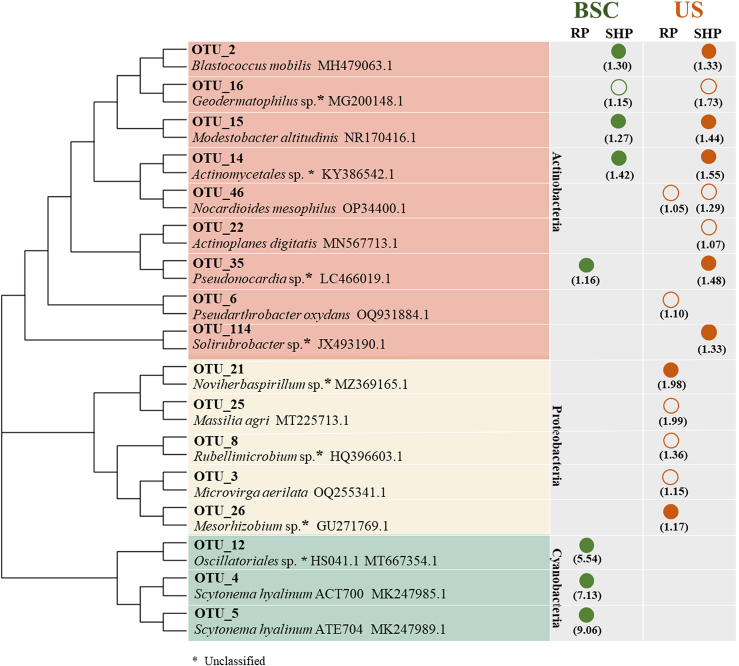
Phylogenetic tree of the active bacteria (^13^C-labelled OTUs) in BSCs and US Solid circles represent HCO_3_^−^-fixer; empty circles represent HCO_3_^−^-transformer. ∗ represents unclassified bacterial lineages. The numbers in parentheses indicate the corresponding REF value for each OTU (threshold of REF >1.0).

OTUs matching criteria (REF >1.0 and relative abundance >1%) were considered as the main active members (OTUs bolded in red, Table S4), and seventeen OTUs were identified. Among them, eleven OTUs were detected in the RP phase, and their phylogenetic tree is visualized in Figure 3. More precisely, only four OTUs were identified in BSCs, and three of them were annotated as Cyanobacteria, including OTU_4 (*Scytonema hyalinum* ACT700, 1.08% of the heavy DNA fraction; 1.07% of the whole bacterial community, same as follows), OTU_5 (*Scytonema hyalinum* ATE704, 1.40% and 1.14%), and OTU_12 (*Oscillatoriales cyanobacterium* HS041.1, 0.52% and 1.32%); while one was annotated as Actinobacteria (OTU_35, *Pseudonocardia* sp, 3.02% and 2.19%).

Table S4. 13C-labeled OTUs and annotation (top 20 OTUs with REF >1.0), related to the identification of 13C-OTUs

In US, seven OTUs were identified as the active members in the RP phase, including OTU_3 (*Microvirga aerilata*, 5.93% and 4.91%), OTU_6 (*Pseudarthrobacter oxydans*, 2.11% and 1.96%), OTU_8 (*Rubellimicrobium* sp., 1.35% and 1.03%), OTU_21 (*Noviherbaspirillum* sp., 1.32% and 1.28%), OTU_25 (*Massilia agri*, 1.25% and 1.02%), OTU_26 (*Mesorhizobium* sp., 1.25% and 1.06%), and OTU_46 (*Nocardioides mesophilus*, 1.09% and 1.15%). Among them, OTU_6 and OTU_46 were annotated as Actinobacteria, while the others were annotated as Proteobacteria.

### Active bacteria responsible for HCO_3_^−^-fixation and carbon cycling in the sustained hydration phase

A total of 8 active OTUs responsible for ^13^C-HCO_3_^-^ assimilation were detected in SHP. In BSCs, they were OTU_2 (*Blastococcus mobilis*, 9.02% and 5.67%), OTU_14 (*Geodermatophilus* sp., 2.38% and 1.86%), OTU_15 (*Modestobacter altitudinis*, 3.65% and 2.09%), and OTU_16 (*Actinomycetales bacterium*, 2.05% and 1.07%), which were all affiliated with Actinobacteria. The identified active OTUs in US were partially consistent with BSCs (OTU_2, 7.68% and 6.88%; OTU_14, 1.93% and 1.34%; OTU_15, 6.06% and 5.48%; and OTU_16, 3.19% and 2.65%), and some others such as OTU_22 (*Actinoplanes digitatis*, 2.13% and 2.06%), OTU_35 (*Pseudonocardia* sp., 1.97% and 1.72%), OTU_46 (*Nocardioides mesophilus*, 1.42% and 1.58%), and OTU_114 (Uncultured *Solirubrobacter* sp., 2.12% and 1.62%) were also identified.

The active bacteria responsible for ^13^C-HCO_3_^-^ assimilation identified above included HCO_3_^−^-fixer and HCO_3_^−^-transformer. Among them, HCO_3_^−^-fixers were OTU_2, OTU_4, OTU_5, OTU_12, OTU_14, OTU_15, OTU_21, OTU_26, OTU_35, and OTU_114 (Figure 3, solid circles). Notably, for unclassified OTUs, their autotrophic potential was referred to their phylogenetic neighbors (Table S3). The active bacteria responsible for HCO_3_^−^-fixation in BSCs accounted for 87.5% of all ^13^C-labeled OTUs, notably higher than that in the US (50.0%). The rest of the OTUs (OTU_3, OTU_6, OTU_8, OTU_16, OTU_22, OTU_25, and OTU_46) were still commonly annotated as heterotrophs. Although novel metabolic potential of HCO_3_^−^-utilization by these well-known heterotrophic bacteria was not excluded, they were classified as HCO_3_^−^-transformers based on the conservative principles in this study (Figure 3, empty circles).

**Figure 4:**
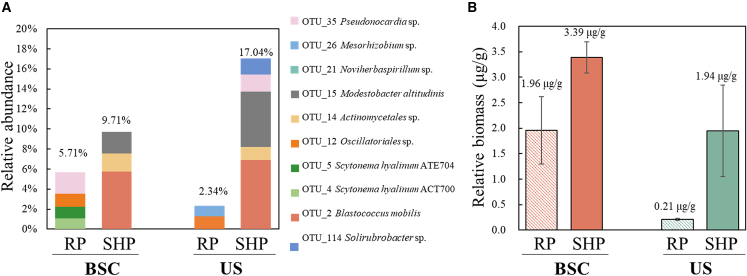
Active ^13^C-labeled bacterial community and biomass (A) Relative abundance of active autotrophs in the whole bacterial community in BSCs and US in the RP and SHP phases. (B) Biomass accumulation in BSCs and US in the RP and SHP phases. US refers to the underlying soil. RP represents the resuscitation phase; SHP represents the sustained hydration phase.

In general, the relative abundance of the active HCO_3_^−^-fixers showed remarkable differences between the RP and SHP phases. More precisely, in BSCs, HCO_3_^−^-fixers only accounted for 5.71% of the whole bacterial community in the RP phase and increased to 9.71% in the SHP phase. A similar trend was also observed in the US, and they increased from 2.34% (RP) to 17.04% (SHP) (Figure 4A), indicating stronger microbial activities in SHP than in the RP phase. Nonetheless, the relative biomass of HCO_3_^−^-fixers in BSCs (RP: 1.96 μg/g, SHP: 3.39 μg/g) was higher than that in US (RP: 0.21 μg/g, SHP: 1.94 μg/g) (Figure 4B), supporting that HCO_3_^−^-fixers were mainly enriched in BSCs rather than US.

### Shifts in HCO_3_^−^ metabolic pathways in BSCs between resuscitation and sustained hydration phases

**Figure 5:**
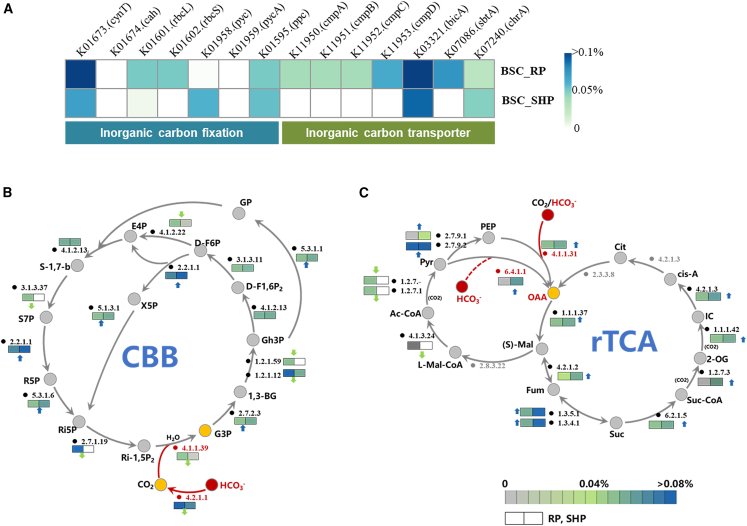
Predicted relative abundances of the key genes by PIRCUSTs (A–C) The relative abundances of key genes encoding carbon transport and fixation. Integrated (B) CBB and (C) rTCA pathways with relative abundance of detected genes. The box on the left represents BSCs in the resuscitation phase (RP), and the right represents BSCs in the sustained hydration phase (SHP). Solid circles represent the metabolites in the two pathways. Red circles represent HCO_3_^−^; yellow circles represent the substances transformed from HCO_3_^−^; gray circles represent others. Black characters represent genes encoding detected enzymes; red represents the key genes encoding inorganic carbon fixation enzymes; gray represents genes encoding non-detected enzymes. (S)-Malate: (S)-Mal; 1,3-Bisphospho-glycerate: 1,3-BG; 2-oxoglutarate: 2-OG; acetyl-coenzyme A: Ac-CoA; cis-Aconitate: *Cis*-A; citrate: Cit; D-Fructose 1,6P2: D-F1,6P2; D-Fructose 6P: D-F6P; Fumarate: Fum; Glyceralde hyde-3P: Gh3P; Glycerate-3P: G3P; Glycerone-P: GP; Isocitrate: IC; L-Malyl-CoA: L-Mal-CoA; oxaloacetate: OAA; phosphoenolpyruvate: PEP; pyruvate: Pyr; Ribose-5P: R5P; Ribulose-1,5P2: Ri-1,5P2; Ribulose-5P: Ri5P; Sedoheptulose-1,7-bisphosphate: S-1,7-b; Sedoheptulose-7P: S7P; Succinate: Suc; Succinyl-CoA: Suc-CoA; Erythrose-4P: E4P; Xylulose-5P: X5P.

PICRUST2 predicted the abundances of key genes in carbon transport and fixation according to the active OTUs responsible for ^13^C-HCO_3_^-^ fixation. The relative abundance of genes encoding some typical bicarbonate transporters is illustrated in Figure 5A, including *cmpABCD* (K11950-53), and some genes belonging to SulP, SbtA, and Chr transporter families (K03321 (*bicA*), K07086 (*sbtA*), and K07240 (*chrA*)). Precisely, all of them were detected in the RP phase, whereas only *bicA* and *chrA* were predicted in the SHP phase. Besides, since carbonic anhydrase (EC:4.2.1.1, CA) catalyzes intracellular or even extracellular carbonate equilibrium (HCO_3_^−^ ⇋ CO_2_), it directly influences the molecular speciation of ^13^C absorbed by the cell and its subsequent utilization. Genes encoding CA (K01673) were remarkably abundant in both RP and SHP phases.

Moreover, for carbon fixation, two main pathways were identified, including the CBB and rTCA cycles (Figures 5B and 5C). Among these pathways, genes encoding 16 and 15 key enzymes were identified in the CBB and rTCA cycles, respectively, and their abundance showed notable differences between the RP and SHP phases. In BSCs, the relative abundance of genes encoding seven enzymes in the CBB cycle decreased significantly in the SHP phase in comparison with the RP phase (Figure 5B), while the abundance of most genes involved in the rTCA cycle increased (Figure 5C).

Precisely, for HCO_3_^−^-fixation in the CBB cycle, HCO_3_^−^ could be transformed to CO_2_ by carbonic anhydrase (EC:4.2.1.1, CA) and then fixed by ribulose-bisphosphate carboxylase (EC:4.1.1.39, RubisCO). Besides, HCO_3_^−^ was involved in the synthesis of oxaloacetate (OAA) by pyruvate carboxylase (EC: 6.4.1.1) and phosphoenolpyruvate carboxylase (EC:4.1.1.31) through the rTCA cycle. In BSCs, the relative abundance of genes encoding RubisCO (K01601, K01602) in the RP phase was notably higher than in the SHP phase, suggesting the important role of the CBB cycle in HCO_3_^−^-fixation in the RP phase. However, the gene encoding phosphoenolpyruvate carboxylase (K01595) was also predicted to exist in the RP phase, and its relative abundance increased in the SHP phase; the genes encoding pyruvate carboxylase (K01958) were only predicted in the SHP phase, emphasizing the potential of the active bacteria using the rTCA cycle to fix HCO_3_^−^ in this scenario (Figure 5A). The above findings were also supported by the predicted relative abundance of carbon fixation pathways that the rTCA pathway was strengthened in bacterial HCO_3_^−^-assimilation from the RP to SHP phases (from 0.09% to 0.83%), whereas the CBB cycle was the opposite (from 0.83% to 0.69%) (Figure S4).

## Discussion

Soil hydrothermal conditions greatly regulate dryland biogeochemical processes,^41^^,^^42^ and soil moisture is widely viewed as a limiting factor in many microbial metabolic processes.^30^^,^^43^^,^^44^ Our work suggested that soil hydration triggered a cascade of microbial activities, driving differences in the appearance of BSCs and physicochemical properties of topsoils between the resuscitation (RP) and sustained hydration (SHP) phases. Precisely, the color of the BSC surface shifted from a slight green to a noticeably darker green in the SHP phase compared to the RP phase (Figure S1), likely due to the recovery and assembly of microbial photosynthetic pigments as previously reported.^45^^,^^46^ Besides, the sprouting mosses also contribute in a non-negligible way to the visual greening of the biocrust surface. The significantly higher TOC contents, sprouting levels of resuscitated mosses, and extractable DNA concentrations were found in SHP (*p* < 0.05, Figure 1B and Table S2), suggesting the notable changes in microenvironmental conditions between these two phases. Previous metagenomic findings suggested that the sources of energy acquisition of resuscitated microorganisms differed across growth periods,^14^^,^^47^ implying the involvement of different participants in inorganic carbon fixation.^48^^,^^49^ However, it is challenging to establish a direct association of inorganic carbon fixation with specific microorganisms based on sequencing data alone. DNA-SIP provides unprecedented insights into the active carbon-fixing microorganisms to unravel the underlying carbon fixation mechanisms in BSCs.

From DNA-SIP results, *B. mobilis*, *M. altitudinis*, *S. hyalinum* (strains ACT700 and ATE704), and some unclassified-proteobacterial, actinobacterial, and cyanobacterial OTUs were identified as the active HCO_3_^−^-fixers (Figure 3, solid circles), which were directly connected to HCO_3_^−^-fixation for the first time. Different types of ^13^C-labeled OTUs demonstrated the diversity of HCO_3_^−^-fixers in BSCs and the underlying soils. In the RP phase, photoautotrophic Cyanobacteria were the dominant HCO_3_^−^-fixers in BSCs, consistent with other studies showing Cyanobacteria as the initial primary producers in BSCs.^13^^,^^50^ The only previously identified terrestrial Cyanobacteria related to HCO_3_^−^-fixation was *Nostoc flagelliforme* affiliated with Nostocales,^51^ consistent with our findings (*S. hyalinum*). Additionally, as nearly all microbial populations resuscitated within minutes or hours after hydration,^14^^,^^49^ non-cyanobacterial autotrophs made a key contribution to HCO_3_^−^-assimilation in the RP and SHP phases. Precisely, the HCO_3_^−^-fixers in the RP phase were affiliated with Actinobacteria (OTU_35, BSCs) and Proteobacteria (OTU_21 and OTU_26, US); while in the SHP phase, they were all annotated as Actinobacteria, with both BSCs and US shared similar contributors (OTU_2, OTU_14, OTU_15, and OTU_35, Figure 3). It was probably explained by the deepened ecological connections and bacterial dispersion between the BSCs and US in the SHP phase under persistent available moisture conditions.^52^^,^^53^^,^^54^ Based on previous DNA-SIP experiments, HCO_3_^−^-utilization by Actinobacteria and Proteobacteria has been demonstrated in soils or other ecosystems (e.g., lakes),^55^^,^^56^ where they are annotated as genera *Corynebacterium, Paucibacter*, *Hydrogenophaga*, *Mycobacterium*, *Ralstonia*, *Ochrobactrum, Sphingomonas*, *Pseudoxanthomonas*, *Burkholderia*, and so forth. Our findings have potentially expanded the inventory of non-cyanobacterial HCO_3_^−^-utilizers. Among Actinobacteria identified here, genomic annotations revealed that both *B*. *mobilis* (OTU_2) and *M*. *altitudinis* (OTU_15) possess a chemolithoautotrophic potential, with mixotrophic characteristics that support their extensive colonization of oligotrophic environments.^57^^,^^58^ Compared to the RP phase, the higher enrichment of Actinobacteria in the SHP phase might be related to the abundant nutrients (TOC: 45.6 g/kg, Figure 1). However, photoautotrophs were scarcely detected in the SHP phase (Table S4). Therefore, our findings reasonably suggested different members contributing to microbial inorganic carbon fixation in the RP and SHP, with the main HCO_3_^−^-fixers switching from photoautotrophs to chemoautotrophs. The absence of cyanobacterial labeling in the SHP phase might relate to the following factors: (1) HCO_3_^−^ assimilation could be diluted by the direct utilization of CO_2_ by Cyanobacteria; (2) Cyanobacteria may undergo functional shifts that move from carbon fixation in oligotrophic conditions to nitrogen fixation in the nutrient-sufficient phase.^59^

The ambient HCO_3_^−^ might be taken up by cells through diverse transport pathways. Particularly, cells possessing CCM mechanisms (such as Cyanobacteria or some Proteobacteria) are reported to exhibit extracellular carbonic anhydrase activity,^18^ suggesting that the ambient HCO_3_^−^ might be converted to CO_2_ outside the cell and then absorbed. Nevertheless, genes encoding bicarbonate transporters were predicted in both RP and SHP phases based on ^13^C-labelled OTUs (Figure 5A), hinting at the potential ability of these cells to actively transport HCO_3_^−^ for subsequent biosynthesis through different carbon fixation pathways. Furthermore, parallel to the changes in the active HCO_3_^−^-fixers in this study, the predicted carbon fixation pathways obtained in BSCs shifted from a CBB-dominant pattern in the RP phase to a rTCA-dominant pattern in the SHP phase (Figure 5). Similar findings were also reported by metagenomic studies.^22^^,^^47^^,^^60^ For example, BSCs from the Tengger Desert (China) showed an increasing contribution of the rTCA pathway and a decreasing role of the CBB and 3HP pathways along with BSC development.^22^ BSCs collected from the Negev Desert (Israel) exhibited higher abundance of RuBisCO genes and microbial carbon fixation rates after wetting in arid and hyper-arid areas than in sub-humid and semi-arid areas.^60^ CBB cycle is reported as the pathway with the highest biomass yield, and photosynthetic Cyanobacteria were most responsible for the enrichment of the CBB pathway in the early-stage BSCs.^61^ They acquire energy through photochemical reactions, with subsequent primary production providing substrates for other biological activities.^62^^,^^63^^,^^64^ However, microorganisms freshly resuscitated from drought were reported to repair macromolecules and replenish reserves coupled with slow growth,^14^^,^^65^ explaining the lower biomass of BSCs in RP (Table S2) and suggesting that microbes might undergo environmental adaptation and substrate accumulation in the RP phase. In addition, the rTCA pathway corresponds to ^13^C-labeled chemoautotrophs in the SHP phase (Figure 3). Particularly, the trace gas (such as H_2_ or CO) oxidation serves as an important energy source for those arid-adapted chemoautotrophs in BSCs reported by previous studies.^57^^,^^58^^,^^60^ Critically, the rTCA pathway was also reported with significantly lower energy demands and higher catalytic efficiency compared to the CBB pathway,^66^^,^^67^ suggesting that bacterial inorganic carbon assimilation in BSCs might shift from a high-yield biosynthesis to an energy-efficient pattern with development.^22^

Additionally, nutrient abundance in the SHP (especially for available nitrogen and phosphorus, Figure S2) might greatly facilitate bacterial proliferation (Figure 4A). Admittedly, besides bacteria, moss spores also resuscitated after hydration, and their germination required days or even weeks.^68^^,^^69^ We observed moss flourish in the SHP (Figure S1), suggesting the difference in response rate between microorganisms and plants (represented by mosses) to hydration. Hence, given the limited net primary production of chemoautotrophs,^27^ the main primary producers in the SHP were plants rather than bacteria.^60^ Interestingly, the active chemoautotrophs in the SHP (*Modestobacter* and *Blastococcus*, Figure 3) were also reported to possess nitrogen fixation potential,^70^^,^^71^ implying the association of ^13^C-labeling with nitrogen fixation processes. Furthermore, bacterial community structure remained stable throughout incubation, showing the unchanged type of BSCs. It might be due to the synchronized proliferation of bacterial phyla (Figure 1E), demonstrating the resilience of the microbial community of BSCs to environmental fluctuations during short-term incubation. Notwithstanding, the dynamics of active HCO_3_^−^-fixers suggested that microbial carbon fixation strategy was flexible and largely responded to resource availability (including nutrients and moisture), rather than BSC types as previously reported.^22^^,^^72^

From the notable spatial change of biomass in different phases (Figure 3), the significantly higher biomass in BSCs than in the US indicated that the BSCs are the primary sites for HCO_3_^−^-fixation, especially the more pronounced accumulation of autotrophic biomass in SHP (Figure 4B). This supported their importance for the primary production of infertile dryland soils.^73^^,^^74^ HCO_3_^−^-transformers identified in this study were mostly enriched in the US, and their ^13^C-labeling might relate to the nutrient cross-feeding between autotrophs and heterotrophs.^75^^,^^76^ Hence, our DNA-SIP results hinted at a spatially top-down transfer of carbon flow from the BSCs to the US. Briefly, dissolved HCO_3_^−^ in soils was assimilated by the active autotrophs in BSCs; the produced organic compounds, e.g., glutamate, dihexose, trihexose, and dihexosylglycerol,^77^ were released and utilized as nutrients by other active microbial members,^78^^,^^79^ particularly for heterotrophs in subsoils by nutrient downward transport.^80^^,^^81^

HCO_3_^−^-fixation of BSCs is an important piece of the jigsaw in the inorganic carbon fixation framework in drylands, especially during the annual wet season windows. Our findings together proposed a nuanced hypothesis on microbial inorganic carbon fixation patterns in BSC during resuscitation and sustained hydration phases. As ^13^C-labeled autotrophs were mostly aerobic bacteria (Table S3), we questioned the widely accepted view that HCO_3_^−^-fixation normally occurs under CO_2_ depletion conditions,^18^ even in anaerobic environments.^82^^,^^83^ Instead, the huge diversity of the active bacterial autotrophs, including Cyanobacteria, Actinobacteria, and Proteobacteria, ensures the functional redundancy and robustness for carbon sequestration under a variety of environmental conditions,^84^^,^^85^ thus enabling a rapid response to resource changes in ecosystems.^86^ More importantly, the potential shift of two key microbial energy production strategies as predicted by PICRUSt (photosynthesis and chemosynthesis) from the RP to SHP phases might represent the dynamics of bacterial ecological roles in BSCs, possibly attributing to the ecological niche differentiation during incubation.^87^^,^^88^

### Conclusion

In this study, we conducted a 14-day hydration incubation for BSCs collected from the Qinghai-Tibetan Plateau, and ^13^C-NaHCO_3_ was used to identify the active bacteria incorporating HCO_3_^−^-fixation in the RP and SHP phases. Hydration significantly triggered microbial resuscitation, and BSCs became more prosperous in SHP compared to the RP phase, including a greener surface, higher topsoil TOC contents, and larger biomass. Our DNA-SIP results confirmed the involvement of *B. mobilis*, *M. altitudinis*, *S. hyalinum* (ACT700 and ATE704), and some unclassified strains in HCO_3_^−^-fixation for the first time. In BSCs, the dominant active HCO_3_^−^-fixers shifted from cyanobacterial photoautotrophs (RP) to actinobacterial chemoautotrophs (SHP), supporting the change in inorganic carbon fixation from the CBB-dominated to the rTCA-dominated pathway. These dynamics were likely explained by the differences in the response rates of autotrophic bacteria and mosses to moisture and the niche differentiation during incubation. Additionally, ^13^C-carbon transformers were detected mostly in the underlying soils, implying carbon flow from BSCs. Our findings unveiled the resuscitated HCO_3_^−^-fixers in BSCs across different growth phases and offered insights into the critical roles of bacteria in carbon fixation in dryland ecosystems.

### Limitations of the study

Several factors may affect the accurate identification of ^13^C-labelled bacteria: (1) a relatively loose REF (>1.0) screening criterion was used, considering that HCO_3_^−^ is not microorganisms’ primary inorganic carbon source, and to expand the detectable diversity of labeled bacteria; (2) given the potential interactions between plants and microorganisms, particularly in the SHP phase, we do not rule out the possibility that plants (mosses) may release ^13^C-labeled organic matter; (3) DNA-SIP requires sufficient isotope labeling to identify the target bacterial taxa and might miss some active carbon-utilizers with low DNA replication rates. In addition, functional predictions of active bacteria were made using PICRUSt 2 rather than through direct measurement. Future work integrating metagenomic approaches could provide more direct functional evidence where feasible.

## Resource availability

### Lead contact

Requests for further information and resources should be directed to and will be fulfilled by the lead contact, Dayi Zhang (zhangdayi@tsinghua.org.cn).

### Materials availability

This study did not generate new unique reagents.

### Data and code availability

• Raw sequencing data have been deposited in the Sequence Read Archive: PRJNA1187533.
• This paper does not report original code.
• Any additional information required to reanalyze the data reported in this paper is available from the lead contact upon request.

## Acknowledgments

The authors gratefully acknowledge the instrumental support provided by the Instrument Sharing Platform in the Department of Environmental Science and Engineering at 10.13039/100031935Xi'an Jiaotong University. Additionally, this study is financially supported by the Key Research and Development Program of Xizang Autonomous Region (no. XZ202001ZY0003G) and the Science and Technology Program of Shenyang (23-407-3-09).

## Author contributions

Y.X.: investigation, data curation, visualization, and writing – original draft preparation. T.T.: methodology. H.L.: investigation. D.Z.: conceptualization, resources, methodology, supervision, writing – original draft preparation, and writing- reviewing and editing.

## Declaration of interests

The authors declare no conflict of interest in this research.

## STAR★Methods

### Key resources table

**Table:** 

REAGENT or RESOURCE	SOURCE	IDENTIFIER
**Biological samples**		

Topsoil samples	A degraded land near Lhasa City	N/A

**Critical commercial assays**		

DNA amplification and sequencing	Guangzhou Magigene Biotechnology Co., Ltd.	https://www.magigene.com

**Deposited data**		

16S rDNA gene raw data	Sequence Read Archive	PRJNA1187533

**Software and algorithms**		

RStudio	Open-source software	https://www.r-project.org/
Excel 2010	Microsoft commercial software	https://www.microsoft.com/
SPSS (v.22.0)	IBM commercial statistical software	https://www.ibm.com/products/spss-statistics
Canoco 5	Commercial software for ecological ordination analysis	https://www.canoco5.com/
MEGA 11	Open-source molecular evolutionary genetics analysis software	https://www.megasoftware.net/
cutadapt (v. 1.14)	Open-source tool for adapter trimming	https://cutadapt.readthedocs.io/
FASTP (v. 0.14.1)	Open-source tool for fast preprocessing of sequencing data	https://github.com/OpenGene/fastp
USEARCH (v10)	Commercial/academic sequence analysis tool	https://www.drive5.com/usearch/
Ribosomal Database Project (RDP) Classifier (v.2.2)	Open-source taxonomic classification tool for 16S rRNA	https://rdp.cme.msu.edu/classifier/classifier.jsp

**Other**		

BLAST (Basic Local Alignment Search Tool	Open-source sequence alignment tool (NCBI)	https://blast.ncbi.nlm.nih.gov/Blast.cgi
PICRUSt2	Open-source bioinformatics software for functional prediction	https://github.com/picrust/picrust2/
SLIVA (v.138.2) database	Open-source ribosomal RNA gene database	https://www.arb-silva.de/
Kyoto Encyclopedia of Gene and Genomes (KEGG)	Open-source database for biological pathways and functions	https://www.genome.jp/kegg/

### Method details

#### BSCs sampling

Lhasa City (China) is located in the center of the Qinghai-Tibetan Plateau. It has an average annual temperature of 5.3°C and annual precipitation of about 400–500 mm,^89^^,^^90^ and the most rainfall occurs in the wet season (June to September). Besides, the semi-arid climate here shapes the landscape with extensive BSC coverage. Topsoil samples (0–1.5 cm, including BSCs and the underlying soils (US)) were collected in November 2021 from a degraded land near Lhasa City (90°48′27.8″ E, 29°22′43.5″ N, altitude of 3550 m) by careful separation from the ground surface using the ring knife and steel knife, sampled into 6 cm sterile polystyrene petri dishes (28 cm^2^ with an average mass of 38.5 g). Soil moisture was generally less than 0.1% (soil hygrometer, ML3 kit, Delta T Ltd., UK), showing a drought condition. Based on visual assessment, the collected BSCs were identified as mixed crusts primarily composed of lichens, with a less presence of mosses. Such BSCs are widely distributed on the Qinghai-Tibetan Plateau. Besides, the average topsoil physicochemical properties were: pH of 8.1, total organic carbon (TOC) of 18.5 g/kg, total nitrogen (TN) of 650.0 mg/kg, and total phosphate (TP) of 986.7 mg/kg. Particularly, the water soluble HCO_3_^−^ was average 68 mg/kg, whereas CO_3_^2−^ was not detected. Samples were transported immediately to the laboratory and stored at 4°C before microcosm incubation.

#### SIP microcosms

Generally, topsoil samples were incubated in a light incubator at 25°C for 14 days (16-h light and 8-h dark per day). To study the active HCO_3_^−^-fixers in the resuscitation phase (gradual recovery from dormancy after hydration) and the sustained hydration phase (continuous hydration) in BSCs, two groups were set. In groups representing the resuscitation phase (RP, days 0–7), original samples (OS) were wetted and incubated for 7 days; in groups of sustained hydration phase (SHP), resuscitated BSCs from the RP groups were kept moistened and further incubated for another 7 days (days 7–14) (Figure S1). In each group, ^13^C-labelled NaHCO_3_ (>98% atom ^13^C, Shanghai ZZBIO Co., LTD) and ^12^C-NaHCO_3_ were used as inorganic carbon sources, respectively. They were added in water (6 mM NaHCO_3_), and then supplemented into samples to 60% field water holding capacity on the first day of incubation and replenished afterward at a rate of 0.3–0.5 mL/day to maintain moisture and supply sufficient isotopic markers. Topsoils of each treatment were incubated in transparent and airtight chambers (3 L) to prevent cross-contamination. They were ventilated for 1 h per day to equilibrate atmospheric pressure and ensure gas exchange. The dilute effect of bacterial fixation of ^13^C-HCO_3_^-^ in the microcosms mainly came from biotic CO_2_ absorption. Each treatment was carried out in three replicates. BSCs and US were separately collected after incubation for DNA extraction and physicochemical property analysis.

#### Physicochemical analysis

Physicochemical variables, including pH, TOC (total organic carbon), TN (total nitrogen), TP (total phosphorus), NH_3_-N (ammonia nitrogen), NO_3_-N (nitrate), and AP (available phosphorus) were measured according to previously reported methods,^91^ which were suggested by the Ministry of Ecology and Environment of China.

#### DNA extraction and ultracentrifugation

DNA was extracted from triplicated 0.25 g aliquots of each BSC and US samples by using the DNeasy PowerSoil Pro Kit (QIAGEN GmbH, Germany) following the manufacturer’s instructions. DNA concentrations and purity were measured using NanoDrop (NanoDrop Technologies, Wilmington, DE, USA). For ultracentrifugation, the replicated DNA samples within each treatment were pooled, and ∼5 μg homogenized DNA was added into a sterile centrifuge tube (5 mL) and mixed with Tris-EDTA (TE, pH 8.0)/CsCl solution to achieve 1.725 g/mL of buoyant density (BD) by AR200 digital refractometer (Reichert, Inc, USA). The solution was further transferred into another 5.1 mL Quick-Seal polyallomer tube (13 × 51 mm, Beckman Colter, Pasadena, CA, USA) with the syringe, added with Tris-EDTA (TE, pH 8.0)/CsCl solution to adjust mass balance, and heat-sealed with a tube topper. The sealed tubes were then placed into a vertical rotor (Vti 65.2, Beckman) for density gradient ultracentrifugation at 45,000 rpm (20 °C) for 48 h. Subsequently, DNA solutions with different BD were separately collected using a fraction recovery system (Beckman Coulter, USA). After measuring the BD value of each fraction, all DNA fractions were purified using Glycogen RNA Grade, and the concentrations were quantified using NanoDrop. Considering the limited DNA concentration for each fraction, the light and heavy fractions were pooled together based on BD comparison for further DNA sequencing. Precisely, for each treatment (including ^12^C-DNA and ^13^C-DNA), the BD value corresponding to the peak of ^13^C-DNA relative abundance exhibited a distinct displacement compared to ^12^C-DNA. The BD value of 1.7274 g/mL was set as the threshold for DNA stratification that light-DNA (fractions with BD values <1.7274 g/mL) and heavy-DNA (fractions with BD values >1.7274 g/mL) fractions were separated for further amplification and sequencing.

#### DNA amplification and sequencing

Amplification and sequencing of light- and heavy-DNA were used to explore the active HCO_3_^−^-fixers, whereas DNA without ultracentrifugation was used to identify the change in bacterial community composition along incubation time. Before amplification, all DNA samples were incorporated and standardized to 25 ng/μL. Primer sets of 515F (5′-GTGCCAGCMGCCGCGGTAA-3′) and 907R (5′-CCGTCAATTCMTTTRAGTTT-3′) were employed to amplify the V4-V5 hypervariable region of bacterial 16S rRNA genes through a standard thermocycling with relevant annealing temperature (55°C) in triplicate.^92^ Amplicons were sequenced on an Illumina Miseq PE 300 by Guangzhou Magigene Biotechnology Co., Ltd. After removing primers using cutadapt (v. 1.14), raw paired-end reads were quality-controlled using FASTP (v. 0.14.1) for sliding-window trimming (window size: 4, required quality: 20), and reads shorter than 100 bp were removed. The paired-end clean reads were merged into raw tags using the fastq_mergepairs command in USEARCH (v10), with a minimum overlap length of 16 bp and allowing up to 5 mismatches in the overlap region. The raw tags were further subjected to sliding-window quality trimming (window size and required mean quality set as before) using FASTP (v. 0.14.1) to generate the final high-quality clean tags. Moreover, on average, 38% of the tags were identified as chimeras and removed, and the remaining tags were merged into operational taxonomic units (OTUs) using the UPARSE method with a similarity threshold of 97%. Taxonomical identification of bacterial 16S rRNA gene was performed using the Ribosomal Database Project (RDP) Classifier (v.2.2),^93^ with an 80% confidence cutoff on the SLIVA (v.138.2) database.^94^

#### Identification of the active HCO_3_^−^-fixation and carbon cycling bacteria

Since ^13^C-labelled bacteria were enriched in the heavy-DNA fractions in the ^13^C-labelled treatment, the relative enrichment factor (REF) was applied to identify these active HCO_3_^−^-fixers, which was calculated in Equation 1.^95^^,^^96^

(Equation 1) REF=(C13−heavy/C13−light)/(C12−heavy/C12−light)

where “^13^C-heavy” and “^13^C-light” represent the relative abundance of OTUs in the heavy- and light-DNA fractions in BSC or US incubated with ^13^C-labelled NaHCO_3_, respectively. “^12^C-heavy” and “^12^C-light” are the relative abundance of OTUs in the heavy- and light-DNA fractions in BSC or US incubated with ^12^C-labelled NaHCO_3_, respectively. OTUs with REF >1.0^96^^,^^97^ and relative abundance >1.0% were identified as the active HCO_3_^−^-fixers. BLAST (Basic Local Alignment Search Tool, https://blast.ncbi.nlm.nih.gov/Blast.cgi) was then applied to search the sequences highly similar to the functional OTUs. The phylogenetic tree of these active ^13^C-labelled OTUs was constructed using MEGA 11 software based on the neighbor-joining method. Additionally, the identification of ^13^C-labelled OTUs annotated as HCO_3_^−^-fixer or HCO_3_^−^-transformer was referenced to previous findings (Table S3). For classified ones, their carbon fixation potential was validated from prior studies reporting their annotated genes or enzymes in their genomes; the potential of unclassified bacterial taxa was hypothetically highlighted according to the phylogenetically closely related species with recorded carbon fixation capabilities.

### Quantification and statistical analysis

Data statistics and bar/line charts were all done on Excel 2010, and the significance was calculated using a double total *t* test performed by SPSS (v.22.0). Principal component analysis (PCA) was visualized by Canoco 5. The relative biomass of the active autotrophs was calculated according to their relative abundance in the whole bacterial communities and DNA concentrations based on 0.25 g sample extracts, described in Equation S1.

(Equation 2) Relativebiomass=RA×C

where RA represents the relative abundance of the autotrophs in the whole bacterial community (%); C represents the concentration of DNA extracted by 0.25 g sample (μg/g). DNA concentrations of each sample are listed in Table S1.

The relative abundance of bacterial enzymes and genes responsible for carbon metabolism was predicted from functional OTUs by PICRUSt2, derived from the KEGG (Kyoto Encyclopedia of Gene and Genomes) catalog.^98^

## Supplemental information

Document S1. Figures S1–S4, Tables S1–S3, and supplemental references
