# Supplementary material for: Change of the active bacteria mediating HCO3--fixation in biological soil crusts using DNA-based stable isotope probing
Source: iScience. 2026 Apr 28;29(6):115898. doi: 10.1016/j.isci.2026.115898 (PMC13207448; doi:10.1016/j.isci.2026.115898)
Supplement: Document S1. Figures S1–S4, Tables S1–S3, and supplemental references [file mmc1.pdf]

iScience, Volume *29*

Supplemental information

Change of the active bacteria mediating HCO_3^--fixation in biological soil crusts using DNA-based stable isotope probing

Yucheng Xie, Huilin Li, Tingting Teng, and Dayi Zhang

Table S1. Detailed BD values corresponding to the heavy-DNA and light-DNA fractions for each sample. Related to the separation of the heavy- and light-fractions after DNA ultracentrifugation

		BSC		US	
		**Light-fraction**	**Heavy-fraction**	**Light-fraction**	**Heavy-fraction**
**RP**	^{12}**C**	≤1.7187	≥1.7274	≤1.7198	≥1.7306
	^{13}**C**	≤1.7209	≥1.7317	≤1.7208	≥1.7317
**SHP**	^{12}**C**	≤1.7273	≥1.7361	≤1.7186	≥1.7284
	^{13}**C**	≤1.7273	≥1.7371	≤1.7219	≥1.7274
**General description**		Light fraction: < 1.7274; heavy fraction: ≥1.7274			

RP represents the resuscitation phase, and SHP represents the sustained hydration phase.

Table S2. DNA concentrations from BSCs and the underlying soils (US). Related to the calculation of the relative abundance of active bacteria in the whole bacterial communities (Figure 4).

**Stage**	**BSC (ng/μL)**		**US (ng/μL)**	
	**RP**	**SHP**	**RP**	**SHP**
**Duplicate 1**	52.8	95.5	19.9	43.7
**Duplicate 2**	96.2	79.7	23.9	21.7
**Duplicate 3**	107.7	86.5	23.5	20.2
Unit conversion (μg/g)				
**Stage**	**BSC (μg/g)**		**US (μg/g)**	
	**RP**	**SHP**	**RP**	**SHP**
**Duplicate 1**	21.12	38.2	7.96	17.48
**Duplicate 2**	38.48	31.88	9.56	8.68
**Duplicate 3**	43.08	34.60	9.40	8.08

RP represents the resuscitation phase, and SHP represents the sustained hydration phase.

Table S3. Main ^{13}C-labelled OTUs and annotation. Related to Figure 3.

	Annotation	Query cover from BLAST	Respiration type	Nutrition type	References
OTU_4	*Scytonema hyalinum* ACT700(MK247985.1)	100%	aerobic	photoautotrophic	[1, 2]
OTU_5	Scytonema hyalinum ATE704(MK247989.1)	100%	aerobic	photoautotrophic	
OTU_12	*Oscillatoriales* cyanobacterium HS041.1 (MT667354.1)	100%	aerobic	photoautotrophic	[3, 4]
OTU_2	*Blastococcus mobilis*(MH479063.1)	100%	aerobic	chemoautotrophic	[5]
OTU_15	*Modestobacter altitudinis*(NR170416.1)	100%	aerobic	chemoautotrophic	[6]
OTU_21	*Noviherbaspirillum* sp.(MZ369165.1)	100%	——	autotrophic*	[7, 8]
OTU_14	Actinomycetales bacterium(KY386542.1)	100%	——	autotrophic*	[9]
OTU_26	Uncultured *Mesorhizobium* sp.(GU271769.1)	100%	——	autotrophic*	[10]
OTU_35	*Pseudonocardia* sp. (LC466019.1)	100%	——	autotrophic*	[11]
OTU_114	Uncultured *Solirubrobacter* sp.(JX493190.1)	100%	——	autotrophic*	Unpublished data
OTU_25	*Massilia agri*(MT225713.1)	100%	aerobic	Heterotrophic*	[12-14]
OTU_6	*Pseudarthrobacter oxydans*(OQ931884.1)	100%	aerobic	heterotrophic	[15]
OTU_46	*Nocardioides mesophilus*(OP34400.1)	100%	aerobic	heterotrophic	[16, 17]
OTU_3	*Microvirga aerilata*(OQ255341.1)	100%	aerobic	heterotrophic	[18-20]
OTU_22	*Actinoplanes digitatis*(MN567713.1)	100%	aerobic	heterotrophic	[21]
OTU_8	Uncultured *Rubellimicrobium* sp.(HQ396603.1)	100%	——	heterotrophic*	[22-24]
OTU_16	*Geodermatophilus* sp.(MG200148.1)	100%	——	heterotrophic*	[25, 26]

-Oxygen requirements of identified bacteria are not provided for unclassified or uncultured strains; * represents the autotrophic/heterotrophic potential owing to their phylogenetic neighbors. References documenting nutrition types for the identified active bacteria are listed in the subsequent column.

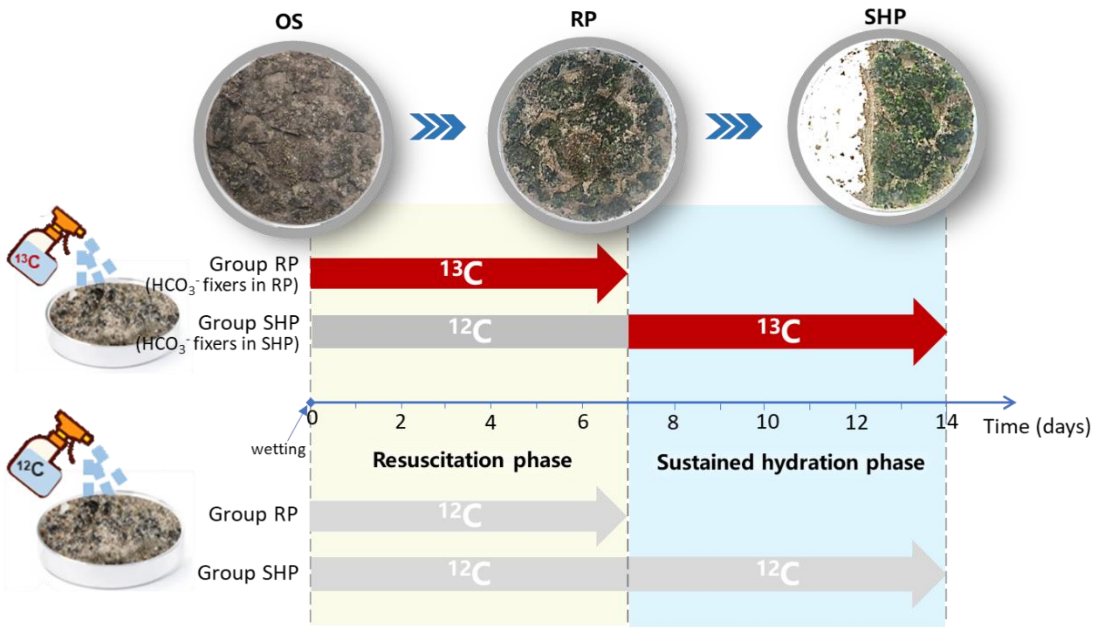

Figure S1. Conceptual diagram of incubation condition. OS represents original BSCs and the underlying soils (US). RP represents the resuscitation phase; SHP represents the sustained hydration phase. Related to the experimental design.

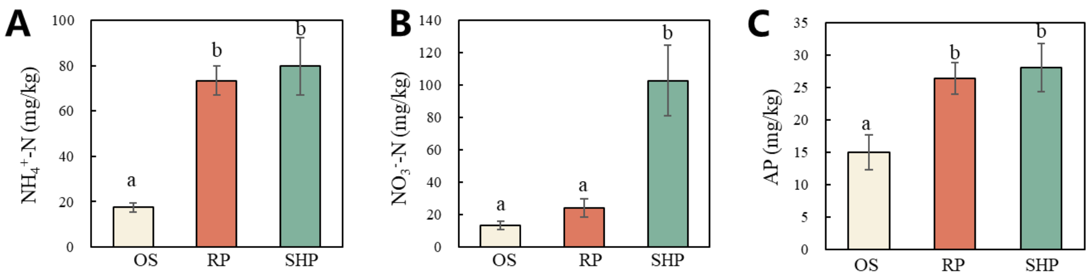

Figure S2. The physicochemical characteristics of topsoil over different periods. Contents of NH_4^+-N (A), NO_3^--N (B), AP (C) in topsoils collected from original sample (OS), RP stage, and SHP stage. RP represents the resuscitation phase, and SHP represents the sustained hydration phase. Related to Figure 1.

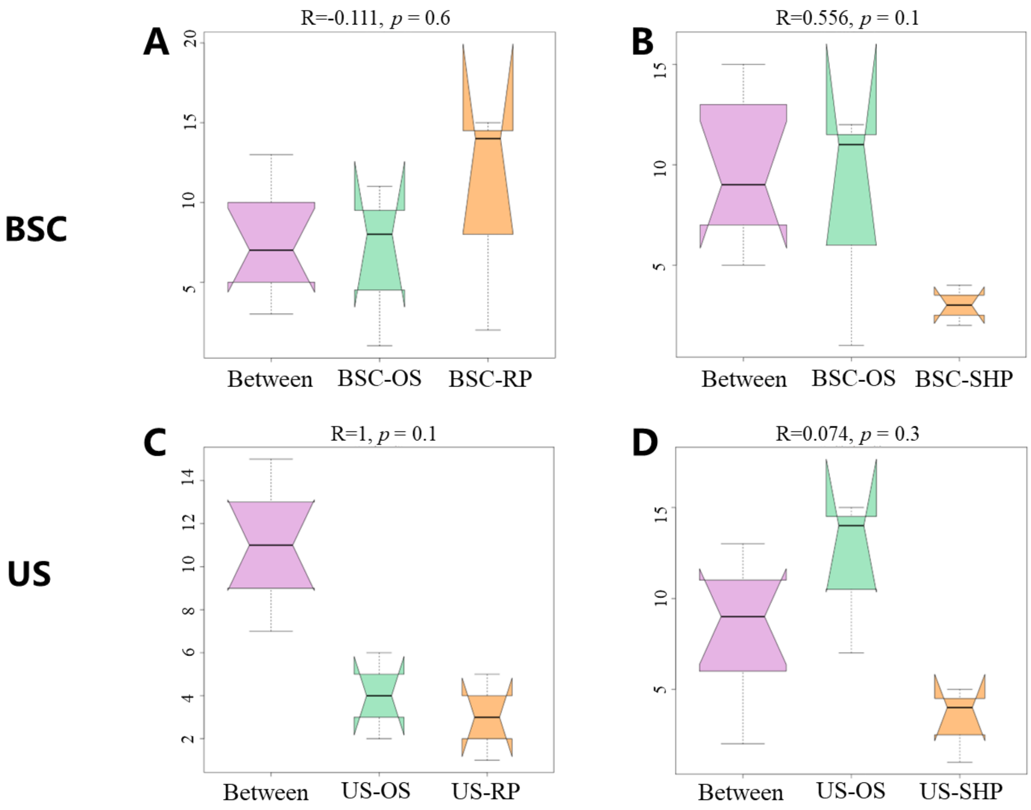

Figure S3. Intergroup differences of bacterial communities under different treatments. (A) ANOSIM of bacterial community structures in BSCs between OS and RP. (B) ANOSIM of bacterial community structures in BSCs between OS and SHP. (C) ANOSIM of bacterial community structures in US between OS and RP. (D) ANOSIM of bacterial community structures in US between OS and SHP. US refers to the underlying soil. OS represents the original sample. RP represents the resuscitation phase, and SHP represents the sustained hydration phase. Related to the assessment of between-group differences in bacterial community structure across different treatments.

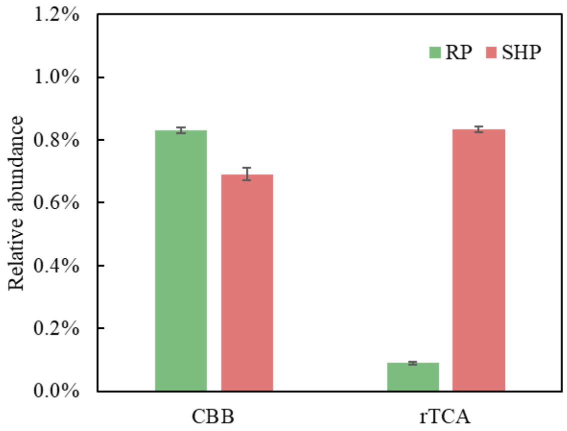

Figure S4. The relative abundance of the main carbon fixation pathways of the active HCO_3^--fixers predicted by PIRCUSTs. Related to the Figure 5.

References

1. Roncero-Ramos, B., Roman, J.R., Acien, G., Canton, Y., (2022). Towards large scale biocrust restoration: producing an efficient and low-cost inoculum of N-fixing cyanobacteria. Science of the Total Environment 848, 157704. https://doi.org/10.1016/j.scitotenv.2022.157704.
2. Roncero-Ramos, B., Angeles Munoz-Martin, M., Chamizo, S., Fernandez-Valbuena, L., Mendoza, D., Perona, E., Canton, Y., Mateo, P., (2019). Polyphasic evaluation of key cyanobacteria in biocrusts from the most arid region in Europe. Peerj 7, e6169. https://doi.org/10.7717/peerj.6169.
3. Milferstedt, K., Kuo-Dahab, W.C., Butler, C.S., Hamelin, J., Abouhend, A.S., Stauch-White, K., McNair, A., Watt, C., Carbajal-Gonzalez, B.I., Dolan, S., et al., (2017). The importance of filamentous Cyanobacteria in the development of oxygenic photogranules. Scientific Reports 7, 17944. https://doi.org/10.1038/s41598-017-16614-9.
4. Moisander, P.H., Daley, M.C., Shoemaker, K.M., Kolte, V., Sharma, G., Garlick, K., (2022). Nitrogen fixation influenced by phosphorus and nitrogen availability in the benthic bloom-forming *Cyanobacterium Hydrocoleum* sp. identified in a temperate marine lagoon. Journal of Phycology 58(3), 377-391. https://doi.org/10.1111/jpy.13244.
5. Montero-Calasanz, M.d.C., Yaramis, A., Rohde, M., Schumann, P., Klenk, H.-P., Meier-Kolthoff, J.P., (2023). Genotype-phenotype correlations within the *Geodermatophilaceae*. Frontiers in Microbiology 13, 1100319.

https://doi.org/10.3389/fmicb.2022.1100319.

6. Golinska, P., Swiecimska, M., Montero-Calasanz, M.d.C., Yaramis, A., Igual, J.M., Bull, A.T., Goodfellow, M., (2020). *Modestobacter altitudinis* sp. nov., a novel actinobacterium isolated from Atacama Desert soil. International Journal of Systematic and Evolutionary Microbiology 70(5), 3513-3527. https://doi.org/10.1099/ijsem.0.004212.

7. Xu, Y., Teng, Y., Dong, X., Wang, X., Zhang, C., Ren, W., Zhao, L., Luo, Y., Greening, C., (2021). Genome-resolved metagenomics reveals how soil bacterial communities respond to elevated H_2 availability. Soil Biology & Biochemistry 163, 108464. https://doi.org/10.1016/j.soilbio.2021.108464.

8. Huang, Y.-M., Straub, D., Kappler, A., Smith, N., Blackwell, N., Kleindienst, S., (2021). A novel enrichment culture highlights core features of microbial networks contributing to autotrophic Fe(II) oxidation coupled to nitrate reduction. Microbial Physiology 31(3), 280-295. https://doi.org/10.1159/000517083.

9. Guo, G., Kong, W., Liu, J., Zhao, J., Du, H., Zhang, X., Xia, P., (2015). Diversity and distribution of autotrophic microbial community along environmental gradients in grassland soils on the Tibetan Plateau. Applied Microbiology and Biotechnology 99(20), 8765-8776. https://doi.org/10.1007/s00253-015-6723-x.

10. Kalloniati, C., Tsikou, D., Lampiri, V., Fotelli, M.N., Rennenberg, H., Chatzipavlidis, I., Fasseas, C., Katinakis, P., Flemetakis, E., (2009). Characterization of a *Mesorhizobium loti* α-type carbonic anhydrase and Its role in symbiotic nitrogen fixation. Journal of Bacteriology 191(8), 2593-2600.

https://doi.org/10.1128/jb.01456-08.

11. Grostern, A., Alvarez-Cohen, L., (2013). RubisCO-based CO_2 fixation and C_1 metabolism in the actinobacterium *Pseudonocardia dioxanivorans* CB1190. Environmental Microbiology 15(11), 3040-3053. https://doi.org/10.1111/1462-2920.12144.

12. Huenninghaus, M., Dibbern, D., Kramer, S., Koller, R., Pausch, J., Schloter-Hai, B., Urich, T., Kandeler, E., Bonkowski, M., Lueders, T., (2019). Disentangling carbon flow across microbial kingdoms in the rhizosphere of maize. Soil Biology & Biochemistry 134, 122-130. https://doi.org/10.1016/j.soilbio.2019.03.007.

13. Zu, D., Wanner, G., Overmann, J., (2008). *Massilia brevitalea* sp nov., a novel betaproteobacterium isolated from lysimeter soil. International Journal of Systematic and Evolutionary Microbiology 58, 1245-1251. https://doi.org/10.1099/ijs.0.65473-0.

14. Chaudhary, D.K., Kim, J., (2017). *Massilia agri* sp nov., isolated from reclaimed grassland soil. International Journal of Systematic and Evolutionary Microbiology 67(8), 2696-2703. https://doi.org/10.1099/ijsem.0.002002.

15. Bushra, R., Uzair, B., Ali, A., Manzoor, S., Abbas, S., Ahmed, I., (2023). Draft genome sequence of a halotolerant plant growth-promoting bacterium *Pseudarthrobacter oxydans* NCCP-2145 isolated from rhizospheric soil of mangrove plant *Avicennia marina*. Electronic Journal of Biotechnology 66, 52-59. https://doi.org/10.1016/j.ejbt.2023.08.003.

16. Zhang, W., Jia, X., Chen, S., Wang, J., Ji, R., Zhao, L., (2020). Response of soil

microbial communities to engineered nanomaterials in presence of maize (Zea mays L.) plants. Environmental Pollution 267. https://doi.org/10.1016/j.envpol.2020.115608.

17. Geng, H., Wang, F., Yan, C., Ma, S., Zhang, Y., Qin, Q., Tian, Z., Liu, R., Chen, H., Zhou, B., et al., (2022). Rhizosphere microbial community composition and survival strategies in oligotrophic and metal(loid) contaminated iron tailings areas. J. Hazard. Mater. 436, 129045. https://doi.org/10.1016/j.jhazmat.2022.129045.

18. Weon, H.-Y., Kwon, S.-W., Son, J.-A., Jo, E.-H., Kim, S.-J., Kim, Y.-S., Kim, B.-Y., Ka, J.-O., (2010). Description of *Microvirga aerophila* sp. nov and *Microvirga aerilata* sp. nov., isolated from air, reclassification of *Balneimonas flocculans* Takeda *et al*. 2004 as *Microvirga flocculans* comb. nov. and emended description of the genus *Microvirga*. International Journal of Systematic and Evolutionary Microbiology 60, 2596-2600. https://doi.org/10.1099/ijs.0.018770-0.

19. Li, Y.-J., Chuang, C.-H., Cheng, W.-C., Chen, S.-H., Chen, W.-L., Lin, Y.-J., Lin, C.-Y., Shih, Y.-h., (2022). A metagenomics study of hexabromocyclododecane degradation with a soil microbial community. Journal of Hazardous Materials 430, 128465. https://doi.org/10.1016/j.jhazmat.2022.128465.

20. Gao, W., Xu, J., Zhao, J., Zhang, H., Ni, Y., Zhao, B., Tebbe, C.C., Zhang, J., Jia, Z., (2020). Prokaryotic community assembly after 40 years of soda solonetz restoration by natural grassland and reclaimed farmland. European Journal of Soil Biology 100, 103213. https://doi.org/10.1016/j.ejsobi.2020.103213.

21. Qu, Z., Bao, X.-d., Xie, Q.-y., Zhao, Y.-x., Yan, B., Dai, H.-f., Chen, H.-Q., (2018).

Actinoplanes sediminis sp nov., isolated from marine sediment. International Journal of Systematic and Evolutionary Microbiology 68(1), 71-75. https://doi.org/10.1099/ijsem.0.002451.

22. Cao, Y.-R., Jiang, Y., Wang, Q., Tang, S.-K., He, W.-X., Xue, Q.-H., Xu, L.-H., Jiang, C.-L., (2010). *Rubellimicrobium roseum* sp nov., a Gram-negative bacterium isolated from the forest soil sample. Antonie Van Leeuwenhoek International Journal of General and Molecular Microbiology 98(3), 389-394. https://doi.org/10.1007/s10482-010-9452-2.
23. Aguilera-Huertas, J., Cuartero, J., Ros, M., Pascual, J.A., Parras-Alcantara, L., Gonzalez-Rosado, M., Ozbolat, O., Zornoza, R., Egea-Cortines, M., Hurtado-Navarro, M., et al., (2023). How binomial (traditional rainfed olive grove-Crocus sativus) crops impact the soil bacterial community and enhance microbial capacities. Journal of Environmental Management 345, 118572. https://doi.org/10.1016/j.jenvman.2023.118572.
24. Fan, G., Zhang, J., Wang, Y., Huang, K., Wang, S., Yao, Y., Luo, J., (2022). Microbial community and nitrogen transformation pathway in bioretention system for stormwater treatment in response to formulated soil medium. Process Safety and Environmental Protection 161, 594-602. https://doi.org/10.1016/j.psep.2022.03.077.
25. Liu, R., Li, K., Zhang, H., Zhu, J., Joshi, D., (2014). Spatial distribution of microbial communities associated with dune landform in the Gurbantunggut Desert, China. Journal of Microbiology 52(11), 898-907. https://doi.org/10.1007/s12275-014-

4075-3.

26. Xie, F., Pathom-aree, W., (2021). Actinobacteria from desert: diversity and biotechnological applications. Frontiers in Microbiology 12, 765531. https://doi.org/10.3389/fmicb.2021.765531.
